# Impact of a pharmacist-led optimization strategy for cerebrovascular pharmacotherapy beyond recanalization in acute ischemic stroke: A pre- and post-intervention study

**DOI:** 10.1371/journal.pone.0357101

**Published:** 2026-08-27

**Authors:** Zhongqiu Zhang, Xincai Zhao, Mengqi Jia, Fei Zhao, Miaomiao Zhou, Yadi Liu, Jiangshan Deng, Xiaoran Cai, Yao Fu, Jianping Zhang, Li Cao, Cheng Guo, Jin Lu

**Affiliations:** 1 Department of Pharmacy, Shanghai Sixth People’s Hospital Affiliated to Shanghai Jiao Tong University School of Medicine, Shanghai, China; 2 Neurology and Genetics Clinical Pharmacy Team, Shanghai Sixth People’s Hospital Affiliated to Shanghai Jiao Tong University School of Medicine, Shanghai, China; 3 Department of Clinical Pharmacy, Shanghai General Hospital, Shanghai Jiao Tong University School of Medicine, Shanghai, China; 4 Department of Neurology, Shanghai Sixth People’s Hospital Affiliated to Shanghai Jiao Tong University School of Medicine, Shanghai, China; 5 Shanghai Neurological Rare Disease Biobank and Precision Diagnostic Technical Service Platform, Shanghai, China; 6 Neurological Disorder Center, Haikou Orthopedic and Diabetes Hospital of Shanghai Sixth People’s Hospital, Haiko, Hainan, China; 7 State Key Laboratory of Neurology and Oncology Drug Development, Nanjing, Jiangsu Province, China; Chinese Academy of Medical Sciences and Peking Union Medical College, CHINA

## Abstract

**Objective:**

Polypharmacy involving cerebrovascular agents is common in acute ischemic stroke, but its clinical and economic value remains unclear. This study evaluated the impact of a pharmacist-led Stroke Pharmacotherapy Optimization Strategy (SPOS) in reducing polypharmacy, drug-related costs, and adverse events while preserving neurological recovery.

**Methods:**

A pre-post study was conducted at a tertiary hospital from June 2024 to April 2025. The control cohort included patients admitted from June to October 2024, and the SPOS cohort included patients admitted from December 2024 to April 2025. SPOS followed the “3-2-1” principle (≤3 non-recanalization cerebrovascular drugs, [NR-CVDs]; ≤ 2 cerebrovascular-related traditional Chinese medicines, [CR-TCMs]) to simplify pharmacotherapy and integrated pharmacist-led medication monitoring, patient education, and individualized adjustments.

**Results:**

A total of 416 patients were included (204 control, 212 SPOS). The SPOS group received significantly fewer NR-CVDs (SPOS group: 3.03 ± 1.18 vs control group: 4.62 ± 1.15; *P* < 0.001) and CR-TCMs (SPOS group: 1.04 ± 0.90 vs control group: 1.93 ± 1.02; *P* < 0.001), without difference in ΔNIHSS or overall neurological recovery. Although the length of hospital stay was similar between groups, total hospitalization costs, drug costs, and the proportion of drug-related expenditures were markedly reduced in the SPOS group (all *P* < 0.001), and the incidence of adverse events was lower in the SPOS group (P = 0.034), with no evident safety signal associated with medication simplification during hospitalization.

**Conclusions:**

Pharmacist-led SPOS in acute ischemic stroke reduced unnecessary cerebrovascular medications and drug costs and was associated with a lower incidence of in-hospital adverse events, without compromising short-term neurological recovery.

## Introduction

Healthcare systems worldwide are facing the growing challenge of rising medical expenditures and the need to ensure the sustainability of health insurance funds [1]. Many countries have implemented reforms emphasizing rational medicine use, value-based healthcare, and standardized evidence-based medication management. Within this evolving healthcare landscape, pharmacists play an increasingly vital role in promoting rational pharmacotherapy [2], improving therapeutic outcomes [3,4], and enhancing the overall value of healthcare delivery [5,6].

Globally, stroke is a leading cause of death and disability, and its burden continues to rise. In China, stroke has become the leading cause of mortality and long-term disability. Among Chinese adults aged 40 years and older in 2020, an estimated 17.8 million individuals were living with stroke (prevalence 2.6%), with 3.4 million incident cases (incidence 505.2 per 100,000 person-years) and 2.3 million stroke-related deaths (mortality 343.4 per 100,000 person-years); ischemic stroke accounted for 86.8% of all incident cases [7]. These figures underscore the substantial clinical and economic imperative to optimize acute stroke care, including its pharmacotherapy.

Polypharmacy is highly prevalent in clinical practice and is particularly prominent among stroke patients. Previous studies have shown that approximately 67.7% of hospitalized ischemic stroke patients experienced drug‑related problems (DRPs) [8]. Another investigation reported that over 91% of stroke patients had at least one potential drug‑drug interaction (pDDI), with polypharmacy (use of more than 10 medications) identified as a significant predictor of high‑severity pDDIs [9]. These findings highlight that polypharmacy is both common and closely associated with impaired medication safety and increased drug‑related cost [10].

In acute ischemic stroke (AIS), the therapeutic benefits of vascular recanalization are limited by narrow time windows, contraindications and reperfusion injury [11–13]. Even after successful recanalization, infarct expansion may continue, adversely impacting neurological recovery [14,15]. Consequently, multiple neuroprotective and cerebrovascular agents such as edaravone dexborneol [16,17], butylphthalide [18], urinary kallidinogenase [19], as well as traditional medicines such as ginkgo biloba extract [20], Qingkailing [21], and Qishiwei Zhenzhu Pill [22,23], have been frequently prescribed. However, the concurrent use of multiple cerebrovascular and neuroprotective agents commonly results in polypharmacy, yet robust evidence on the clinical effectiveness, safety, and pharmacoeconomic impact of such combination therapies remains limited. Moreover, polypharmacy increases the risk of drug‑drug interactions, adverse drug reactions, and higher healthcare costs, and is a major contributor to DRPs in AIS. These issues underscore the need for a more systematic approach to pharmacotherapy management in AIS.

In response to these challenges, we developed the Stroke Pharmacotherapy Optimization Strategy (SPOS), a pharmacist‑led framework designed to reduce unnecessary polypharmacy, improve patient safety, and enhance healthcare value. SPOS simplifies cerebrovascular drugs excluding intravenous thrombolysis, antiplatelet therapy, anticoagulation, and fibrinolysis (non-recanalization cerebrovascular drugs, NR-CVDs) based on the “3-2-1” principle: each patient receives ≤3 NR-CVDs and ≤2 cerebrovascular-related traditional Chinese medicines (CR-TCMs), with individualized adjustment (“1 patient”) under pharmacist supervision. By integrating pharmaceutical care, clinical decision support, and patient education, SPOS aims to rationalize therapy and improve patient outcomes. Therefore, this study aimed to evaluate the clinical effectiveness, economic impact, and safety of SPOS compared with usual care in patients with acute ischemic stroke, to provide evidence supporting pharmacist-led optimization in stroke pharmacotherapy. The “3-2-1” principle represents an empirically derived, institution-developed framework for medication stewardship rather than a guideline-recommended or prospectively validated prescribing threshold.

## Materials and methods

### Study design

This retrospective pre-post comparative study was conducted at Shanghai Sixth People’s Hospital between June 2024 and April 2025. Patients were assigned to the pre-intervention (control) or post-intervention (SPOS) cohort according to their admission period. The pre-intervention cohort included patients admitted from June to October 2024 who received standard medical management, whereas the post-intervention cohort included patients admitted from December 2024 to April 2025 after implementation of the pharmacist-led SPOS.

A one-month transition period (November 2024) was incorporated into the study design to allow complete implementation and stabilization of the SPOS protocol before enrollment of the post-intervention cohort. The study protocol, eligibility criteria, outcome definitions, and data collection procedures remained unchanged throughout both study periods. During the study period, no major changes occurred in the hospital formulary, institutional stroke management pathway, or DRG/DIP payment policies.

### Participants

Patients admitted with AIS during the study period were screened. Inclusion criteria: (1) age ≥ 18 years; (2) diagnosis of AIS confirmed by clinical presentation and imaging; (3) presence of acute onset focal neurological deficits (such as unilateral limb weakness, numbness, speech disturbance, or facial palsy), or persistent neurological deficit ≥24 hours even without a clearly identified lesion; and (4) brain CT or MRI excluding intracerebral hemorrhage. Exclusion criteria: (1) severe infection or psychiatric disorder; (2) active malignancy or immunodeficiency; (3) pregnancy, lactation, or known hypersensitivity; (4) severe hepatic or renal dysfunction; (5) other critical illnesses that could affect outcome evaluation or pharmacoeconomic analysis; and (6) patients who self-discharged before receiving systematic treatment. Baseline characteristics, including TOAST classification and National Institutes of Health Stroke Scale (NIHSS) scores on admission, were recorded. All inclusion and exclusion criteria were predefined before data extraction and applied uniformly to both cohorts; patients were excluded solely because key variables required for the predefined analyses were missing, independent of disease severity, clinical outcomes, medication response, or costs.

### Interventions

Both groups received standard medical management according to the Chinese Clinical Pathway for Ischemic Stroke (2016 Edition), including vascular recanalization (intravenous thrombolysis, antiplatelet therapy, anticoagulation, fibrinolysis, and endovascular intervention), neuroprotection, management of comorbidities (hypertension, hyperglycemia, hyperlipidemia), treatment of complications (cerebral edema, elevated intracranial pressure, seizures, infections), early nutritional support, rehabilitation, and individualized secondary prevention. NR-CVDs and CR-TCMs were initiated within 24 hours of admission.

**Fig 1:**
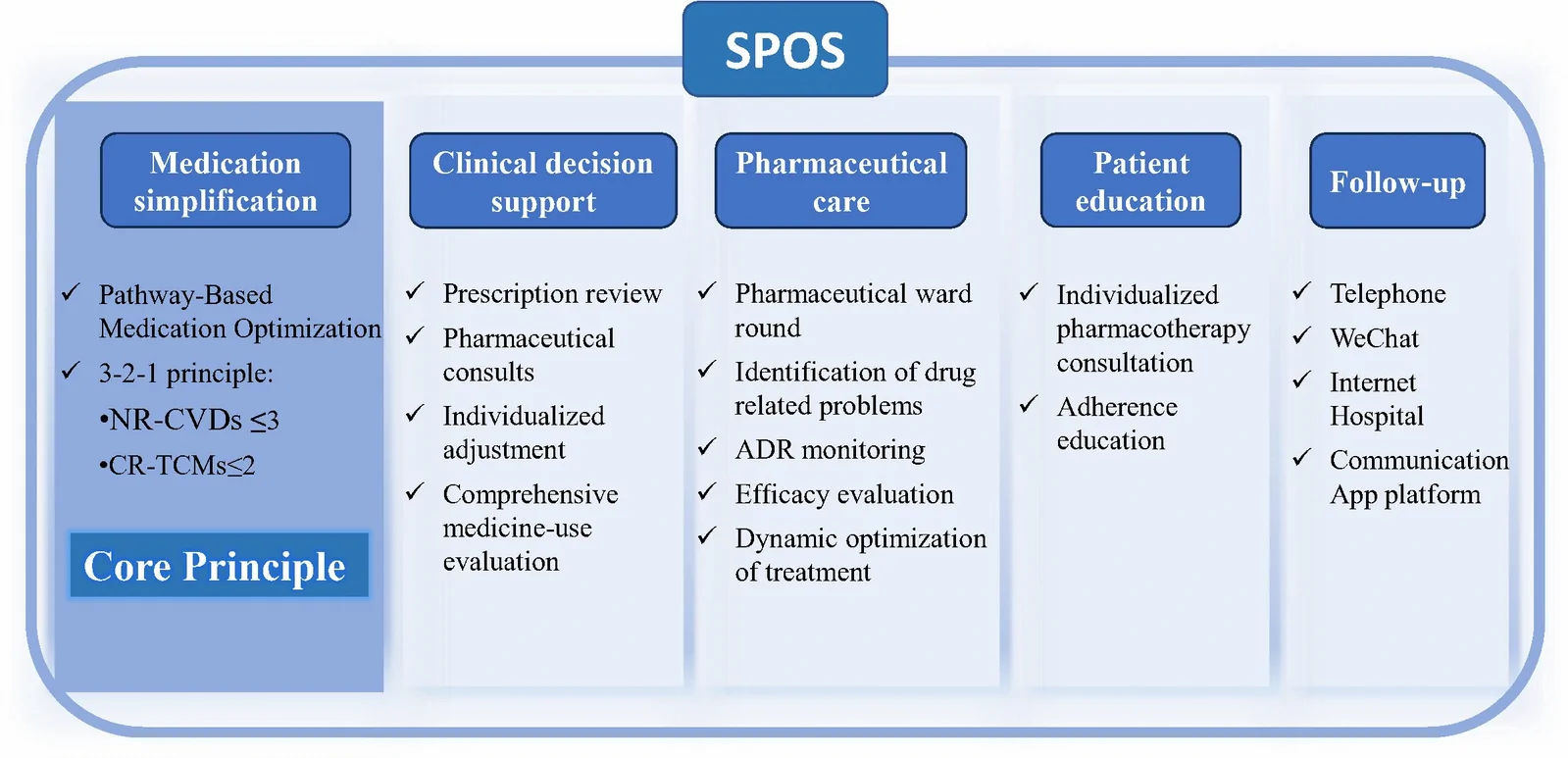
The model of stroke pharmacotherapy optimization strategy. The model is centered on the core principle of medication simplification and integrates clinical decision support, pharmaceutical care, patient education, and follow-up. **Abbreviations**: SPOS: Stroke Pharmacotherapy Optimization Strategy; NR-CVDs: non-recanalization cerebrovascular drugs (cerebrovascular drugs excluding intravenous thrombolysis, antiplatelet therapy, anticoagulation, fibrinolysis); CR-TCMs: cerebrovascular-related traditional Chinese medicines.

From November 2024 onward, a pharmacist-led SPOS was implemented based on standard care. SPOS focused on optimizing NR-CVDs according to the “3-2-1” principle: each patient was prescribed no more than three NR-CVDs, with no more than two CR-TCMs, aiming to minimize unnecessary polypharmacy. Clinical pharmacists provided decision support, patient education, and comprehensive pharmaceutical care throughout hospitalization, including continuous monitoring and follow-up (Fig 1). It should be emphasized that the “3-2-1” principle was developed empirically at our institution as a pragmatic framework for pharmacist-led medication stewardship, rather than as a guideline-recommended or prospectively validated prescribing threshold; it served as the intervention framework in this real-world study. The specific NR-CVDs and CR-TCMs eligible for use, together with their recommended dosing and average daily cost, are listed in S1 Table. Within this framework, clinical pharmacists prioritized agents with relatively stronger supporting evidence according to stroke subtype and individual patient characteristics, avoided concomitant use of agents with overlapping pharmacological mechanisms whenever possible, and individualized therapy based on the patient’s clinical condition rather than applying a rigid numerical rule.

### Data collection

Patient demographic and clinical information were collected, including TOAST classification, pre-existing comorbidities, stroke onset time at admission, and vascular recanalization methods. NIHSS scores were assessed within 24 hours of admission and prior to discharge. For patients who died during hospitalization, a NIHSS score of 42 was assigned at discharge to reflect maximal neurological impairment. The number of NR-CVDs and CR-TCMs used during hospitalization was also recorded.

### Outcomes

The health outcomes were the change in NIHSS score from admission to discharge (△NIHSS) and clinical outcome (improvement, deterioration, or death). Improvement was defined as △NIHSS <0 or, based on joint assessment by physicians and pharmacists, as a reduction in stroke-related symptoms at discharge compared with admission. Deterioration was defined as △NIHSS >0 or, according to clinical judgment by physicians and pharmacists, as worsening of stroke-related symptoms at discharge relative to admission. Incidence of adverse events was recorded as a safety endpoint.

Economic outcomes included length of hospital stay, the number of NR-CVDs and CR-TCMs initiated within 24 hours of admission, total hospitalization costs, drug costs, CR-TCMs costs, and proportion of drug costs. Currency conversion from Chinese Yuan (CNY) to US dollars ($) was based on an exchange rate of 7.0.

### Sample size calculation

The required sample size was estimated to detect a clinically meaningful difference in the total hospitalization cost of AIS between the two groups. Based on previous studies [10], the expected mean difference was 163, with a standard deviation of 496. Using a two-sided α of 0.05 and a statistical power of 90%, the sample size was calculated with the t-test and the corresponding non-centrality parameter, indicating that at least 100 participants were required. The sample size calculation was performed using the online calculator available at https://sample-size.net/sample-size-study-paired-t-test/. Because the calculation was powered for the primary economic endpoint, the study was not adequately powered to detect differences in infrequent safety outcomes such as specific adverse events or mortality.

### Statistical analysis

Statistical analyses were performed using SPSS version 26.0. Categorical variables are presented as counts and percentages, and group comparisons were conducted using the Pearson chi-square test. Continuous variables with a normal distribution were presented as mean ± standard deviation (SD) and compared using independent-samples t-tests. Non-normally distributed continuous variables were reported as median (interquartile range, IQR) and compared using the Mann–Whitney *U* test. All tests were two-tailed, and *p* < 0.05 was considered statistically significant.

### Ethics approval and consent to participate

This study was conducted in accordance with the Declaration of Helsinki and was approved by the Ethics Committee of Shanghai Sixth People’s Hospital (Approval No. 2025-KY-180). Although this was a retrospective study, all participants had signed a general informed consent form upon admission, authorizing the use of their clinical data for research purposes. The data were accessed and collected for research purposes from September 1, 2025, to December 30, 2025. The authors had access to information that could identify individual participants during the data collection phase to ensure accuracy; however, all data were de-identified and anonymized prior to statistical analysis.

## Results

### Baseline characteristics

**Fig 2:**
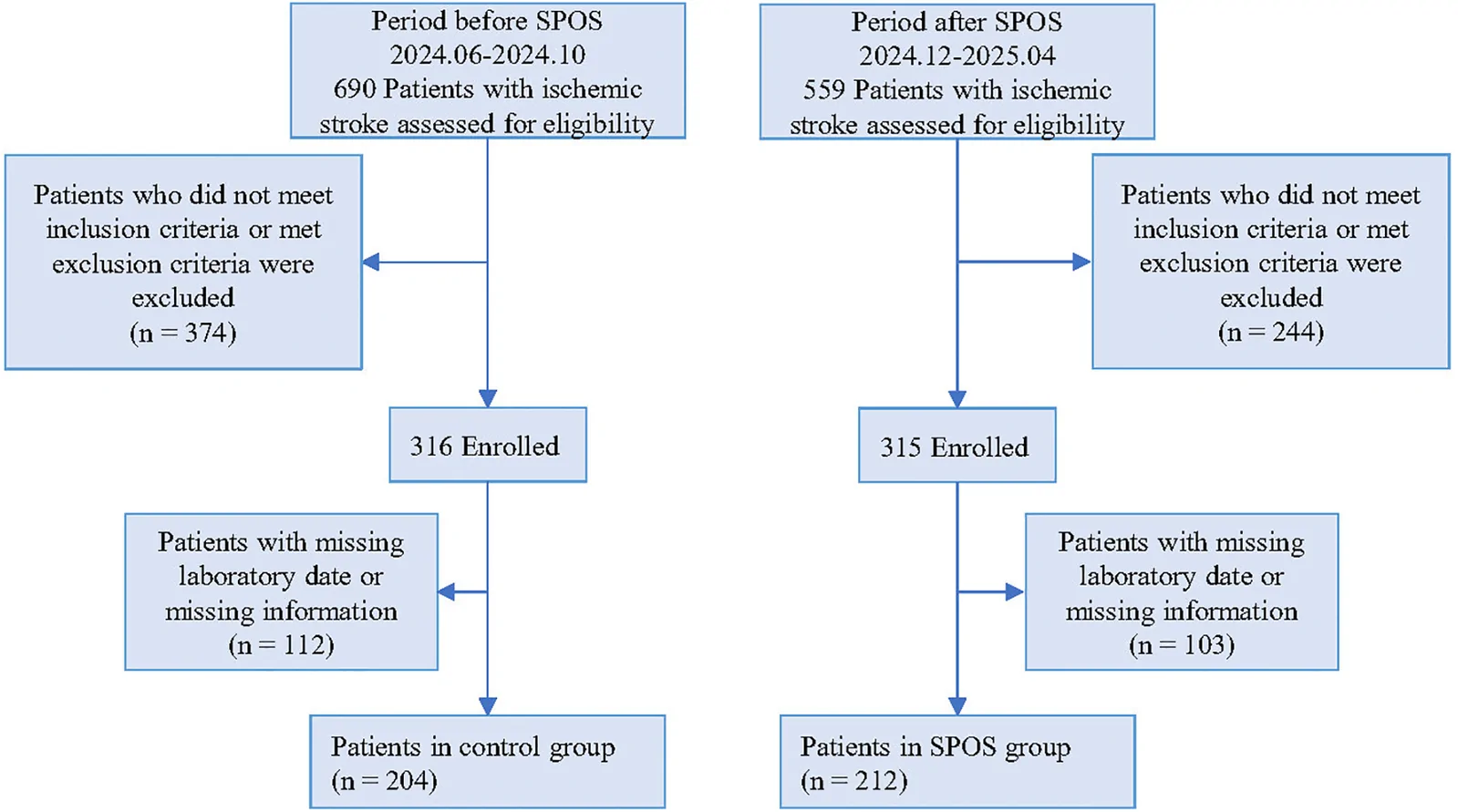
Flowchart. A total of 416 participants were allocated for analysis.

Between June 2024 and April 2025, a total of 1,249 patients with ischemic stroke were screened, including 690 patients during the pre-intervention period and 559 during the post-intervention period. After applying the inclusion and exclusion criteria, 631 patients were eligible for enrollment (316 in the pre-intervention group and 315 in the SPOS group). Among them, 215 patients (112 in the pre-intervention cohort and 103 in the SPOS cohort) were excluded because of incomplete laboratory or clinical information required for the predefined analyses; the numbers of excluded patients were similar between cohorts, and exclusions were unrelated to disease severity, clinical outcomes, or medication costs. Consequently, 416 patients were included in the final analysis: 204 in the pre-intervention group (control group) and 212 in the SPOS group. The patient selection flowchart is shown in Fig 2.

Baseline demographic and clinical characteristics of patients admitted before and after SPOS implementation are shown in Table 1. Overall, patient profiles were similar across the two study periods. The mean age of the 416 patients was 70.5 years, and 65.1% were male. Major comorbidities included hypertension (78.6%), diabetes mellitus (38.5%), atrial fibrillation (13.2%), and a prior history of cerebrovascular disease (27.7%). Among the patients, 67.3% were admitted within 48 hours of stroke onset. Intravenous thrombolysis was administered to 7.0% of patients, while 13.9% underwent endovascular intervention. The mean NIHSS score at admission was 4, with a median score of 2, and most cases were classified as large-artery atherosclerosis or small-artery occlusion.

**Table 1:** Baseline characteristics.

Factor	Control group (204)	SPOS group (212)	*p* value
**Age (y), MEAN ± SD**	71.26 ± 11.37	69.75 ± 12.46	0.195 ^a^
**Sex, N (%)**			0.983 ^b^
Male	133(65.2)	138(65.1)	
Female	71(34.8)	74(34.9)	
**BMI (kg/m** ^**2**^ **), MEAN±SD**	24.54 ± 3.72	24.59 ± 3.14	0.881^a^
**Hypertension, N (%)**	160(78.4)	167(78.8)	0.932 ^b^
**Diabetes, N (%)**	85(41.7)	75(35.4)	0.187 ^b^
**Hyperlipidemia, N (%)**	10(4.9)	20(9.4)	0.074 ^b^
**Atrial fibrillation, N (%)**	26(12.7)	29(13.7)	0.779 ^b^
**Admitted within 48 hours of stroke onset, N (%)**	129(63.2)	151(71.2)	0.082 ^b^
**Prior history of cerebrovascular disease, N (%)**	54(26.5)	61(28.8)	0.600 ^b^
**NIHSS score at admission, MEDIAN [IQR]**	2.0[1, 4]	2.0[0, 5]	0.576 ^c^
**Intravenous thrombolysis, N (%)**	11(5.4)	18(8.5)	0.215 ^b^
**Endovascular intervention, N (%)**	25(12.3)	33(15.6)	0.330 ^b^
**TOAST classification, N (%)**			0.096 ^b^
Large-artery atherosclerosis	129(63.2)	119(56.1)	
Small artery occlusion	59(28.9)	68(32.1)	
Cardioembolism	16(7.8)	20(9.4)	
Other determined etiology	0(0)	0(0)	
Undetermined etiology	0(0)	5(2.4)	

a: Independent *t*-test; b: Pearson chi-square test; c: Mann-Whitney *U* test.

Abbreviations: BMI, body mass index.

### Health outcomes

Health outcomes are summarized in Table 2. Mortality was comparable between the SPOS group (2.4%) and control group (2.0%). Rates of clinical improvement and deterioration were also similar between groups. For the primary clinical outcome, the change in NIHSS score (△NIHSS) in the SPOS group 0.0[−2.0, 0.0] was comparable to that in the control group −0.5[−2.0, 0.0].

**Table 2:** Health outcomes.

Factor	Control group (204)	SPOS group (212)	*p* value
**Clinical outcome**			0.444 ^a^
Death, N (%)	4(2.0)	5(2.4)	
Deterioration, N (%)	8(3.9)	4(1.9)	
Improvement, N (%)	192(94.1)	203(95.8)	
**△NIHSS, MEDIAN [IQR]**	−0.5[−2.0, 0.0]	0.0[−2.0, 0.0]	0.978 ^b^

a: Pearson chi-square test; b: Mann-Whitney *U* test.

Abbreviations: ΔNIHSS, change in NIHSS score from admission to discharge.

Deterioration was defined as △NIHSS > 0 or, according to clinical judgment by physicians and pharmacists, a worsening of stroke-related symptoms at discharge compared with admission.

Improvement was defined as △NIHSS < 0 or, based on joint assessment by physicians and pharmacists, a reduction in stroke-related symptoms at discharge compared with admission.

### Economic outcomes

As shown in Table 3, no significant differences were observed in length of hospital stay (SPOS group: 7.96 ± 3.33 days *vs*. control group: 8.14 ± 2.96 days) or total number of medications used (SPOS group: 19.72 ± 10.64 *vs*. control group: 20.43 ± 8.29). However, the SPOS group received significantly fewer NR-CVDs (SPOS group: 3.03 ± 1.18 *vs*. control group: 4.62 ± 1.15, *p* < 0.001) and CR-TCMs (SPOS group: 1.04 ± 0.90 *vs*. control group: 1.93 ± 1.02, *p* < 0.001) compared with the control group.

**Table 3:** Economic outcomes.

Factor	Control group (204)	SPOS group (212)	*p* value
**Length of hospital stay (days), MEAN ± SD**	8.14 ± 2.96	7.96 ± 3.33	0.550 ^a^
**Total number of medications used, MEAN ± SD**	20.43 ± 8.29	19.72 ± 10.64	0.447 ^a^
**Number of NR-CVDs, MEAN ± SD**	4.62 ± 1.15	3.03 ± 1.18	<0.001 ^a^
**Number of CR-TCMs, MEAN ± SD**	1.93 ± 1.02	1.04 ± 0.90	<0.001 ^a^
**Total hospitalization costs ($), MEDIAN [IQR]**	2267.86 [1781.50, 3226.89]	1803.63 [1513.46, 2637.90]	<0.001 ^b^
**Drug cost ($), MEDIAN [IQR]**	886.43 [684.18, 1215.21]	649.37 [455.21, 830.68]	<0.001 ^b^
**CR-TCMs cost ($), MEDIAN [IQR]**	145.93 [76.96, 233.00]	151.04 [99.30, 210.86]	0.775 ^b^
**Proportion of drug costs (%), MEAN ± SD**	36.35 ± 11.97	29.76 ± 11.11	<0.001 ^a^

a: Independent *t*-test; b: Mann-Whitney *U* test.

Abbreviations: NR-CVDs, non-recanalization cerebrovascular drugs; CR-TCMs, cerebrovascular-related traditional Chinese medicines.

Total hospitalization costs were significantly lower in the SPOS group ($1,803.63 [1,513.46, 2,637.90]) than in the control group ($2,267.86 [1,781.50, 3,226.89]). Meanwhile, drug cost was reduced in the SPOS group ($649.37 [455.21, 830.68] *vs*. $886.43 [684.18, 1,215.21], *p* < 0.001), accompanied by a reduction in the proportion of drug-related costs (SPOS group: 29.76 ± 11.11 *vs*. control group: 36.35 ± 11.97, *p* < 0.001).

### Subgroup analysis

To further assess whether baseline stroke severity influenced the treatment response, we conducted subgroup analyses based on baseline NIHSS scores and onset-to-admission time (Table 4). The stratified analysis, including mild (1–4), moderate (5–15), and moderate-to-severe stroke (16–42), revealed no statistically significant differences in ΔNIHSS between the SPOS and control groups. Similarly, no significant differences were observed when patients were stratified by time from stroke symptom onset to hospital admission (both ≤48 h and >48 h).

**Table 4:** Subgroup analysis of the change in NIHSS score (ΔNIHSS) from admission to discharge.

Factor	Control group		SPOS group		*p* value
	N	MEDIAN [IQR]	N	MEDIAN [IQR]	
**By baseline NIHSS score**					
1 ~ 4	122	−1.0[−1.0, 0.0]	97	−1.0[−2.0, 0.0]	0.346 ^a^
5 ~ 15	36	−3.0[−5.0, 0.0]	43	−2.0[−7.0, 0.0]	0.618 ^a^
16 ~ 42	13	−3.0[−15.5, 2.5]	17	−4.0[−15.5, 0.0]	0.834 ^a^
**By onset to admission**					
≤48 h	129	−1.0[−2.0, 0.0]	151	0.0 [−2.0, 0.0]	0.993 ^a^
>48 h	75	0.0[−2.0, 0.0]	61	0.0[−1.0, 0.0]	0.597 ^a^

a: Mann-Whitney *U* test.

### Safety outcomes

The incidence of adverse events was significantly lower in the SPOS group compared with the control group (p = 0.034). Adverse events in the SPOS group included one case each of gastrointestinal bleeding, diarrhea, dermatitis, and dizziness. In the control group, adverse events included cerebral hemorrhage (1 case), diarrhea (2 cases), dermatitis (2 cases), dizziness (3 cases), drug-induced liver injury (1 case), and hypotension (3 cases) (Table 5). Overall, no evident safety signal associated with medication simplification was observed during hospitalization in the SPOS group; however, given the small number of events, this finding should be interpreted cautiously and not overstated as a definitive safety benefit.

**Table 5:** Safety outcomes.

		control group	SPOS group	*p* value
**Adverse events, N**		12	4	0.034 ^a^
Type of adverse event	Cerebral hemorrhage	1	0	
	Gastrointestinal bleeding	0	1	
	Diarrhea	2	1	
	Dermatitis	2	1	
	Dizziness	3	1	
	Drug-induced liver injury	1	0	
	Hypotension	3	0	

a: Pearson chi-square test.

## Discussion

In this retrospective pre-post comparative study, we developed and evaluated the pharmacist-led SPOS, an institution-specific medication stewardship framework designed to optimize the use of non-recanalization cerebrovascular drugs during acute ischemic stroke hospitalization. Rather than serving as a guideline-recommended prescribing threshold, the “3-2-1” principle represents an empirically developed framework to support rational medication optimization through pharmacist-led multidisciplinary management. By integrating individualized medication review, clinical decision support, and pharmaceutical care into routine practice, SPOS aims to reduce unnecessary polypharmacy while promoting more efficient and evidence-informed pharmacotherapy.

The present study demonstrates that implementation of SPOS significantly reduced the use of NR-CVDs and CR-TCMs without compromising short-term neurological recovery. Length of hospital stay, changes in NIHSS scores, and other in-hospital clinical outcomes remained comparable between the two groups, whereas medication utilization and overall drug expenditure were significantly reduced following implementation of SPOS. It is noteworthy that although the number of prescribed CR-TCMs decreased significantly after implementation of SPOS, CR-TCMs expenditure did not differ significantly between groups. This apparent discrepancy reflects the different dimensions captured by medication counts and expenditure. The primary objective of SPOS was to reduce potentially unnecessary or low-value CR-TCMs prescribing rather than to uniformly decrease medication costs. Moreover, because CR-TCMs costs exhibited a markedly skewed distribution, expenditure was summarized using the median (interquartile range), which is relatively insensitive to a small number of patients with high medication expenditures. Consequently, the reduction in CR-TCMs utilization was not accompanied by a statistically significant difference in median CR-TCMs expenditure.

Although fewer in-hospital adverse events were observed in the SPOS group, the limited number of events precludes definitive conclusions regarding safety. Therefore, the current findings should be interpreted as indicating that medication simplification was not associated with an evident deterioration in short-term inpatient safety rather than demonstrating a confirmed safety benefit. Collectively, these findings suggest that pharmacist-led medication optimization can improve the efficiency of medication utilization during acute ischemic stroke hospitalization while maintaining comparable short-term clinical outcomes. This strategy is particularly relevant in the context of ongoing healthcare payment reform and policies promoting rational medication use and value-based healthcare in China [24–26].

With rapidly increasing healthcare expenditures, drug affordability has become a key challenge for the Chinese healthcare system [26,27]. Pharmacoeconomic evaluation and comprehensive clinical assessment are increasingly applied to optimize prescriptions and reduce costs [1,28]. A growing body of evidence demonstrates that pharmacist-led interventions improve both clinical and economic outcomes across diverse healthcare settings [28–34]. Meta-analyses show that such interventions reduce drug expenditure, hospitalization costs, prescription errors, and adverse events [29]. Beyond traditional medication review, structured physician–pharmacist collaborative prescribing models have emerged as an effective approach to optimizing pharmacotherapy in high-risk patients. For example, Kiracı et al. [34] demonstrated that clinical pharmacist participation significantly improved adherence to guideline-recommended venous thromboembolism prophylaxis, increasing the rate of optimal thromboprophylaxis from 45.4% to 59.2% compared with education alone, highlighting the additional value of active pharmacist involvement in clinical decision-making beyond educational interventions. Similarly, Hale et al. [31] reported that collaborative pharmacist prescribing for venous thromboembolism prophylaxis generated healthcare cost savings while improving quality-adjusted life years, supporting the clinical and economic value of multidisciplinary pharmacotherapy optimization. In addition, pharmacist-led digital monitoring programs in the UK significantly reduced gastrointestinal bleeding [30], while prospective cohort study at West China Hospital found that pharmaceutical care reduced adverse drug events and medication errors, promoted rational use, shortened ICU stays, and lowered treatment costs, demonstrating clear cost-effectiveness [32]. Another study from Xiangya Hospital showed that a collaborative physician-pharmacist pain management model improved clinical outcomes while superior cost-effectiveness [33]. Collectively, these lines of evidence show that pharmacist interventions optimize resource allocation while enhancing both clinical and economic value across disease areas and care models.

In stroke management, previous studies have mainly focused on secondary prevention and long-term care, with pharmacist interventions targeting adherence improvement [35], early identification of high-risk factors such as atrial fibrillation [36], optimization of blood pressure and lipid control [37] identification of drug-related problems [8], medication education [38], and long-term follow-up [39]. These interventions primarily aim to enhance long-term adherence, reduce recurrence risk, and improve long-term outcomes [35–41]. However, the impact of acute-phase cerebrovascular drug type and quantity on short-term outcomes remains underexplored. The novelty of this study lies in shifting pharmacist intervention to the acute phase, and implementing a dynamic SPOS model that integrates reduction of unnecessary polypharmacy, decision support, continuous pharmaceutical care, and patient education. Our findings suggest that reducing unnecessary use of multiple NR-CVDs and CR-TCMs during the acute phase did not compromise short-term neurological recovery while reducing medication burden and overall drug expenditure. No evident deterioration in inpatient safety was observed following implementation of the SPOS strategy; however, given the limited number of adverse events, definitive conclusions regarding safety cannot be drawn. These findings support the feasibility of pharmacist-led medication optimization for promoting more rational pharmacotherapy during acute ischemic stroke hospitalization.

Of course, the therapeutic efficacy of NR-CVDs has been well supported by existing evidence. Edaravone Dexborneol by reducing oxidative stress, reducing cerebral edema, and delaying neuronal death, thereby improving clinical outcomes [16,17]. Urinary kallidinogenase exerts neuroprotective effects by releasing vasoactive kinins through kininogen cleavage, regulating blood pressure, enhancing collateral circulation, improving cerebral blood flow and perfusion, and promoting angiogenesis [19]. Butylphthalide increases local cerebral blood flow, improves microcirculation in the ischemic area [42], and enhances cerebral autoregulation [18], leading to a reduction in neurological deficits and short-term mortality [42]. Ginkgo biloba extract has been shown to increase cerebral blood flow and ameliorate post-stroke cognitive impairment [20]. The Tibetan medicine Qishiwei Zhenzhu pills are traditionally believed to promote circulation and restore consciousness [22], while Qingkailing injection may alleviate oxidative stress and inflammatory responses, thereby protecting neuronal survival [21]. However, these data largely come from single-drug studies, and evidence supporting synergistic effects from polypharmacy is limited; current guidelines do not recommend multiple overlapping agents [15,43]. In our trial, patients received an average of 4.6 NR-CVDs, including nearly 2 CR-TCMs, highlighting the prevalence of polypharmacy. Implementation of SPOS reduced drug numbers and adverse events without compromising efficacy, supporting the rationale for minimizing unnecessary polypharmacy to enhance safety and economic value.

This study also highlights the expanding role of pharmacists in modern stroke care. Within the SPOS framework, pharmacists lead efforts to reduce unnecessary polypharmacy, minimize adverse drug reactions, and lower medication costs, thereby directly enhancing the safety, efficiency, and value of stroke management. By integrating clinical expertise with patient-centered pharmaceutical care, pharmacists ensure rational medication use and promote sustainable healthcare delivery. The SPOS model demonstrates how pharmacist-led interventions can transform stroke pharmacotherapy toward a precision- and value-based paradigm, underscoring the essential contribution of pharmacists to high-quality, cost-effective care.

This study has several limitations that should be considered when interpreting the findings. First, the retrospective pre-post design without randomization, concurrent parallel control, or blinding of clinicians, pharmacists, or outcome assessors is susceptible to temporal bias and residual confounding; therefore, the observed reductions in medication use, costs, and adverse events cannot be attributed solely to the SPOS intervention. Although baseline characteristics were generally balanced between cohorts (Table 1) and no major changes occurred in the hospital formulary, stroke care pathway, or DRG/DIP policies during the study period, we cannot fully exclude the influence of concurrent changes in routine practice. Second, the “3-2-1” principle was developed empirically at our institution as a pragmatic medication-stewardship framework and has not yet been validated through prospective dose-ranging studies, Delphi expert consensus, or multicenter trials; its generalizability as a prescribing threshold therefore remains uncertain. Third, all outcomes were assessed during the index hospitalization, capturing only short-term neurological recovery (ΔNIHSS at discharge) and in-hospital adverse events; the absence of post-discharge follow-up (e.g., 30-day or 90-day recurrence, readmission, mRS scores, or long-term adverse drug reactions) precludes any conclusion regarding long-term effectiveness or safety, and our conclusions are accordingly limited to the inpatient setting. Fourth, although the sample size was adequate for the primary economic endpoint, the study was underpowered to detect differences in infrequent safety outcomes such as specific adverse events or mortality; the lower incidence of adverse events observed in the SPOS group should therefore be interpreted cautiously and not overstated as a definitive safety benefit. Fifth, the economic analysis reported aggregate hospitalization and drug costs without category-specific breakdowns for individual NR-CVDs or CR-TCMs agents, limiting our ability to quantify the relative contribution of each medication class to the observed cost reduction. Finally, as a single-center retrospective study based on routinely collected electronic medical records, incomplete documentation and the predefined exclusion of patients with missing key variables may introduce selection bias and reduce generalizability; the numbers of excluded patients were, however, similar between cohorts (112 vs. 103), and exclusions were unrelated to study outcomes. Future studies incorporating micro-costing approaches and formal sensitivity analyses are warranted to evaluate the economic performance of the SPOS strategy under different healthcare payment systems.

In summary, this study introduces the pharmacist-led SPOS for patients with acute ischemic stroke, demonstrating its capacity to streamline medication use and optimize healthcare resource allocation within the inpatient setting. Although short-term neurological outcomes were not significantly altered, SPOS effectively reduced unnecessary polypharmacy, was associated with a lower incidence of in-hospital adverse events, decreased drug expenditures, and improved overall treatment efficiency. Beyond its clinical impact, SPOS represents an innovative, pharmacist-driven framework that aligns with China’s ongoing healthcare payment reforms and the global shift toward value-based care. By redefining the pharmacist’s role from supportive participation to proactive leadership in therapeutic decision-making, this model provides a scalable paradigm for precision and sustainable stroke pharmacotherapy, although further validation through multicenter, randomized controlled trials with extended follow-up is required.

## Conclusion

Within the inpatient setting, our pharmacist-led SPOS to reduce unnecessary non-recanalization cerebrovascular drug use demonstrated no compromise in short-term neurological recovery reducing unnecessary polypharmacy, with no evident deterioration in inpatient safety, and lowering overall drug expenditures. Distinct from conventional deprescribing interventions, SPOS integrates a disease-specific, pharmacist-developed framework that standardizes cerebrovascular drug use beyond recanalization therapy. This innovative, pharmacist-driven model provides a pragmatic and potentially scalable framework for value-based stroke management. SPOS highlights the pivotal role of clinical pharmacists in supporting more structured and rational pharmacotherapy for acute ischemic stroke; nonetheless, further validation through multicenter, randomized controlled trials with long-term outcome follow-up is required.

## Supporting information

S1 Table Classification of non-recanalization cerebrovascular drugs (NR-CVDs) included in the SPOS strategy. This table summarizes all 16 NR-CVDs included in the SPOS intervention. Among these agents, Drugs 6–16 were classified as cerebrovascular-related traditional Chinese medicines (CR-TCMs).
(DOCX)
