# Supplementary material for: Impact of a pharmacist-led optimization strategy for cerebrovascular pharmacotherapy beyond recanalization in acute ischemic stroke: A pre- and post-intervention study
Source: PLoS One. 2026 Aug 27;21(8):e0357101. doi: 10.1371/journal.pone.0357101 (PMC13521402; doi:10.1371/journal.pone.0357101)
Supplement: Supplementary file 1 [file pone.0357101.s001.docx]

**Supplementary Table S1. Classification of non-recanalization cerebrovascular drugs (NR-CVDs) included in the SPOS strategy.**

This table summarizes all 16 NR-CVDs included in the SPOS intervention. Among these agents, Drugs 6–16 were classified as cerebrovascular-related traditional Chinese medicines (CR-TCMs).

| **No.** | **Drug name** | **Recommended dose and route** | **Specification** | **Average daily cost** |
| --- | --- | --- | --- | --- |
| 1 | Betahistine hydrochloride injection | Intravenous infusion, 30 mg, once daily | 10 mg/2 mL/vial | 5.01 |
| 2 | Butylphthalide and sodium chloride injection | Intravenous infusion, 25 mg, twice daily | 100 mL:25 mg/bottle | 29.19 |
| 3 | Edaravone dexborneol concentrated solution for injection | Intravenous infusion, 15 mL, twice daily | 5 mL:10 mg:2.5 mg/vial | 25.44 |
| 4 | Urinary kallidinogenase for injection | Intravenous infusion, 0.15 PNA units, once daily | 0.15 PNA units/bottle | 14.29 |
| 5 | Ginkgo diterpene lactone meglumine injection | Intravenous infusion, 5 mL, once daily | 5 mL:25 mg/vial | 13.39 |
| 6 | Dan Deng Tong Nao soft capsules | Oral, 4 capsules per administration, three times daily | 0.55 g x 72 capsules/bottle | 6.38 |
| 7 | Danhong injection | Intravenous infusion, 40 mL, twice daily | 10 mL/vial | 19.34 |
| 8 | Duzhi pill | Oral, 5 g per administration, twice daily | 200 pills/box | 1.81 |
| 9 | Peiyuan Tongnao capsules | Oral, 3 capsules per administration, three times daily | 0.6 g x 45 capsules/bottle | 2.34 |
| 10 | Qingkailing injection | Intravenous infusion, 20 mL, once daily | 2 mL x 10 vials/box | 24.00 |
| 11 | Shuxuening injection | Intravenous infusion, 20 mL, once daily | 10 mL/vial | 28.51 |
| 12 | Xingnaojing injection | Intravenous infusion, 4 mL, once daily | 10 mL x 2 vials/box | 18.74 |
| 13 | Ginkgolide injection | Intravenous infusion, 5 vials per administration, once daily | 2 mL:10 mg/vial | 14.06 |
| 14 | Ginkgo leaf extract injection | Intravenous infusion, 5 vials, once daily | 17.5 mg/5 mL/vial | 8.00 |
| 15 | Salvianolic acid for injection | Intravenous infusion, 100 mg, once daily | 100 mg/vial | 7.77 |
| 16 | Xueshuantong injection | Intravenous injection, 150 mg, once daily | 150 mg/vial | 1.93 |
